# Chalcones Display Anti-NLRP3 Inflammasome Activity in Macrophages through Inhibition of Both Priming and Activation Steps—Structure-Activity-Relationship and Mechanism Studies

**DOI:** 10.3390/molecules25245960

**Published:** 2020-12-16

**Authors:** Wohn-Jenn Leu, Jung-Chun Chu, Jui-Ling Hsu, Chi-Min Du, Yi-Huei Jiang, Lih-Ching Hsu, Wei-Jan Huang, Jih-Hwa Guh

**Affiliations:** 1 School of Pharmacy, College of Medicine, National Taiwan University, No. 33, Linsen S. Rd, Taipei 100, Taiwan; r00423018@ntu.edu.tw (W.-J.L.); d97423004@ntu.edu.tw (J.-L.H.); r07423013@ntu.edu.tw (C.-M.D.); r07423015@ntu.edu.tw (Y.-H.J.); lhsu@ntu.edu.tw (L.-C.H.); 2 Graduate Institute of Pharmacognosy, Taipei Medical University, No. 250, Wuxing St., Taipei 110, Taiwan; sk9090666@gmail.com; 3 Department of Pharmacy, New Taipei Municipal TuCheng Hospital, Chang Gung Memorial Hospital, New Taipei City 236, Taiwan

**Keywords:** chalcone, NLRP3 inflammasome, structure-activity relationship, NF-ĸB, ATP, K^+^ efflux

## Abstract

Chalcones are responsible for biological activity throughout fruits, vegetables, and medicinal plants in preventing and treating a variety of inflammation-related diseases. However, their structure-activity relationship (SAR) in inhibiting inflammasome activation has not been explored. We synthesized numerous chalcones and determined their SAR on lipopolysaccharide (LPS)-primed ATP-induced NLRP3 inflammasome activation. 11Cha1 displayed good inhibitory activity on release reaction of caspase-1, IL-1β, and IL-18. It significantly inhibited LPS-induced phosphorylation and proteolytic degradation of IĸB-α and nuclear translocation of NF-ĸB, but had little effect on mitogen-activated protein kinases (MAPKs) activities. Furthermore, 11Cha1 blocked LPS-induced up-regulation of NLRP3, pro-caspase-1, ASC, IL-18, and IL-1β, indicating the suppression on priming step of inflammasome activation. ASC dimerization and oligomerization are considered to be direct evidence for inflammasome activation. 11Cha1 profoundly inhibited ATP-induced formation of ASC dimers, trimers, and oligomers, and the assembly of ASC, pro-caspase-1, and NLRP3 in inflammasome formation. Decrease of intracellular K^+^ levels is the common cellular activity elicited by all NLRP3 inflammasome activators. 11Cha1 substantially diminished ATP-mediated K^+^ efflux, confirming the anti-NLRP3 inflammasome activity of 11Cha1. In summary, the SAR of chalcone derivatives in anti-inflammasome activities was examined. Besides, 11Cha1 inhibited both priming and activation steps of NLRP3 inflammasome activation. It inhibited NF-ĸB activation and subsequently suppressed the up-regulation of NLRP3 inflammasome components including NLRP3, ASC, pro-caspase-1, pro-IL-18, and pro-IL-1β. Next, 11Cha1 blocked ATP-mediated K^+^ efflux and suppressed the assembly and activation of NLRP3 inflammasome, leading to the inhibition of caspase-1 activation and proteolytic cleavage, maturation, and secretion of IL-1β and IL-18.

## 1. Introduction

Chalcones and flavonoids, which are various compounds found naturally throughout fruits and vegetables, are always responsible for biological activity and therapeutic efficacy of herbs in treating and preventing a variety of diseases, such as cancer, atherosclerosis, diabetes, hepatotoxicity, bacterial infection, and inflammation [1,2,3,4]. There are thousands of studies reporting that chalcones and flavonoids display biological activities in diverse in vitro and in vivo models through regulating multiple signaling pathways, including mitogen-activated protein kinases [MAPKs, e.g., Erk1/2, p38 MAPK and c-Jun N-terminal kinase (JNK)], nuclear factor-ĸB (NF-ĸB), phosphoinositide 3-kinase (PI3K)/Akt/mammalian target of rapamycin (mTOR), Janus kinase (JAK)/signal transducers and activators of transcription (STAT), cyclic nucleotide dependent protein kinases, intracellular calcium mobilization, and oxidative stress proteins [1,2,3,4,5,6,7]. The fundamental capability of these compounds is the inhibition of inflammation, which has been linked to most of the diseases. In recent decades, inflammasome has been discovered to be cytosolic multiprotein oligomers responsible for the activation of inflammatory responses [8]. The NOD-, LRR-, and pyrin domain-containing protein 3 (NLRP3) inflammasome, a multimeric protein complex consisting of a sensor NLRP3, an adaptor apoptosis-associated speck-like protein containing a CARD (ASC) and an effector caspase-1, triggers an inflammatory form of cell death named pyroptosis and induces the secretion of proinflammatory cytokines IL-1β and IL-18. Activation of NLRP3 inflammasome in macrophages needs two sequential steps: priming (signal 1) and activation (signal 2). The priming step is to induce the upregulation of NLRP3, caspase-1, and pro-IL-1β expression. The transcriptional upregulation can be triggered through the recognition of pathogen-associated molecular patterns (PAMPs) or damage-associated molecular patterns (DAMPs) that bind pattern recognition receptors or through cytokines (e.g., TNF-α and IL-1β) that induce NF-ĸB activation and gene transcription. Activation step can be induced by PAMPs and DAMPs, leading to NLRP3 inflammasome assembly and caspase-1 activation which mediates IL-1β and IL-18 secretion and pyroptosis [8,9,10,11].

A lot of studies provide evidence that natural flavonoids display biological activities through targeting both priming and activation steps of inflammasome, such as inhibition of NF-ĸB activation, decreased both mRNA and protein expressions of NLRP3 inflammasome components, and disruption of NLRP3 assembly [12]. These studies support that NLRP3 inflammasome may serve as a crucial therapeutic target for anti-inflammation and related diseases. In contrast, several studies have elucidated the effect of chalcones on the inhibition of NLRP3 inflammasome activation. It was reported that *trans*-chalcone inhibits monosodium urate (MSU) crystals-induced pro-inflammatory cytokine production. The inhibition of NF-ĸB activation was responsible for suppressing the expression of inflammasome components. *Trans*-chalcone also blocked MSU-mediated IL-1β release in lipopolysaccharide (LPS)-primed macrophages [13]. Isoliquiritigenin, another chalcone compound with multiple pharmacological properties including antioxidant, anti-inflammation, and modulation of GABAergic synaptic transmission [14], inhibited MSU, nigericin, and adenosine triphosphate (ATP)-induced NLRP3 inflammasome activation in bone marrow-derived macrophages, leading to the suppression of caspase-1 and IL-1β production [15]. Several potential chalcones were also reported to inhibit alcohol/LPS-induced liver steatosis, LPS/d-galactosamine-induced hepatotoxicity, dextran sulfate sodium-induced ulcerative colitis, and Propionibacterium acnes-induced skin inflammation [16,17,18,19]. Because of the therapeutic potential, several active chalcone derivatives have been generated [20]. These studies suggest that chalcone compounds can be potential candidates in anti-NLRP3 inflammasome research.

We have designed and synthesized dozens of chalcone and flavonoid compounds. After the screening of anti-NLRP3 inflammasome activity, we have found several potential chalcone derivatives that potently inhibited LPS-primed ATP-induced caspase-1 and IL-1β production. The mechanism of anti-NLRP3 inflammasome, including the involvement of NF-ĸB activation, NLRP3-ASC assembly and ASC dimerization, K^+^ efflux, and several cellular molecules, has been clearly elucidated to discover the potential of chalcone derivatives.

## 2. Results

### 2.1. Detection of Caspase-1 p20 Fragment was Performed to Screen Chalcone Derivatives with Anti-NLRP3 Inflammasome Activity

**Figure 1:**
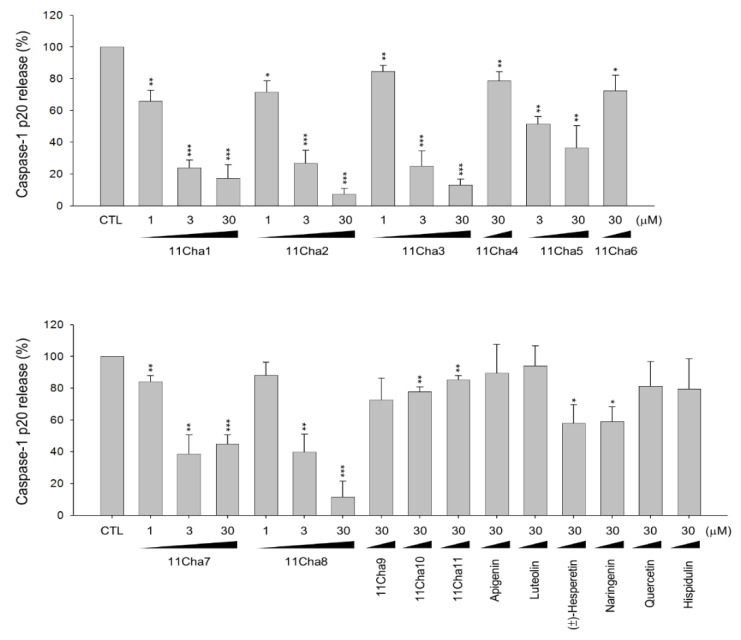
Effects of chalcone derivatives and flavonoids on the inhibition of lipopolysaccharide (LPS)-primed ATP-induced pro-caspase-1 processing in differentiated THP-1 cells. THP-1 cells were seeded and differentiated by 50 nM PMA for 48 h. Then, the cells were primed with 1 μg/mL LPS for 4 h and were pre-treated with or without the indicated compound for 30 min and then treated with 5 mM ATP for another 2 h. After treatment, the concentrations of p20 caspase-1 subunit in the medium were determined. Data are expressed as mean±SEM of three to four independent experiments. * *P* < 0.05, ** *P* < 0.01, and *** *P* < 0.001 compared with compound-free control.

LPS priming followed by exogenously applied ATP stimulates the activation of P2X_7_ receptor and is well recognized as a reliable model in studying NLRP3 inflammasome. In the present work, the model was applied in THP-1 monocytic leukemic cells, which were differentiated into macrophages by phorbol 12-myristate 13-acetate (PMA) to screen anti-NLRP3 inflammasome activity of various chalcone derivatives. We examined the processing of pro-caspase-1 into the p20 subunit, one of the key hallmarks in inducing caspase-1 activity in inflammasome activation. The data in Figure 1 demonstrated that some of the chalcone derivatives displayed anti-inflammasome activities. The data in Table 1 demonstrated that the inhibitory ability of pro-caspase-1 processing was varied with the introduction of different functional group in ring A and B. Less hindered groups on ring A such as hydroxyl, methoxyl, and/or methoxymethoxyl (MOM) (e.g., 11Cha1, 11Cha2, and 11Cha3) increased the activity. However, the bulky benzyl (Bn) and iodine group on either ring A or B (e.g., 11Cha4, 11Cha6, 11Cha9, 11Cha10, and 11Cha11) decreased the activity. Compound 11Cha1 exhibited activity comparable to 11Cha2, suggesting methoxyl and MOM group equally contributed to activity. Notably, the inhibitory activities of these active chalcone derivatives on pro-caspase-1 processing were superior to those flavonoids such as apigenin, luteolin, (±)-hesperetin, naringenin, quercetin, and hispidulin (Table 1). Since 11Cha1 showed the strongest activity among the chalcone derivatives with an IC_50_ of 1.5 µM, the mechanistic study in anti-inflammasome ability was performed.

### 2.2. 11Cha1 Inhibits the Release of Caspase-1 p20 Fragment, IL-1β, and IL-18

**Figure 2:**
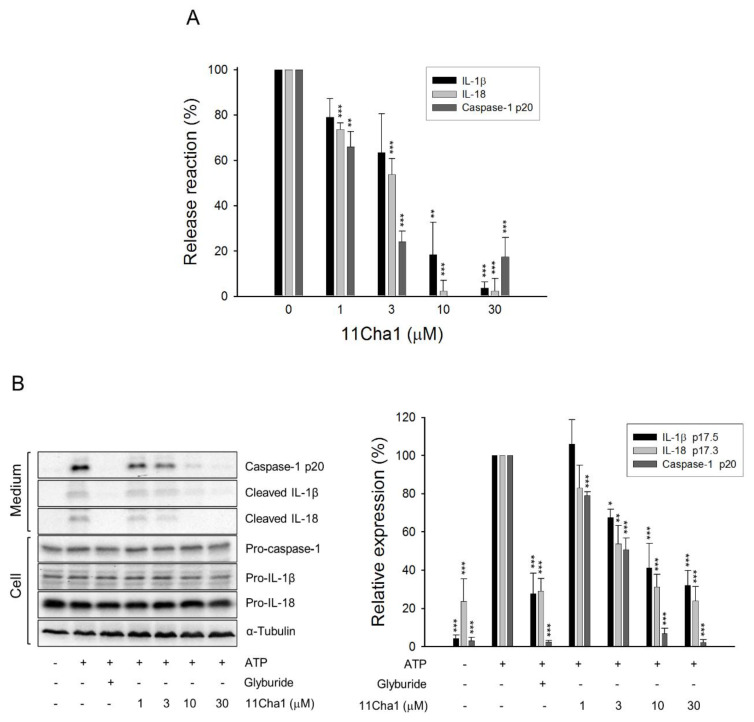
Effects of 11Cha1 on the release reaction and protein expressions of caspase-1 and cytokines. THP-1 cells were seeded and differentiated by 50 nM PMA for 48 h and then primed with 1 μg/mL LPS for 4 h. The cells were pre-treated with or without the indicated agent (200 µM glyburide as the positive control) for 30 min before a two-hour exposure to 5 mM ATP. After treatment, the concentrations (**A**) and protein expressions (**B**) of p20 caspase-1 subunit and cytokines in the medium were determined. Data are expressed as mean±SEM of three independent experiments. One-way ANOVA by Newman Keuls post hoc test was used. * *P* < 0.05, ** *P* < 0.01, and *** *P* < 0.001 compared with ATP-treated control. The analyses of *P* (ANOVA) indicated *P* < 0.001 in all tests.

Upon NLRP3 inflammasome activation, autoproteolysis of pro-caspase-1 produced large (p20) and small (p10) cleavage fragments of the catalytically active enzymes, leading to the cleavage of pro-IL-1β and pro-IL-18 into their secreted active forms [9,21,22]. The detection of release reaction in the medium using ELISA system demonstrated that 11Cha1, by itself without cytotoxic effect (Supplementary Materials Figure S1), caused a concentration-dependent inhibition of ATP-induced caspase-1 activation and the release of IL-1β and IL-18 in LPS-primed macrophages with IC_50_ values of 1.5 ± 0.1, 4.9 ± 1.8, and 3.2 ± 0.5 µM, respectively (Figure 2A). Western blotting analysis further substantiated that 11Cha1 significantly inhibited ATP-mediated release reaction in LPS-primed macrophages with IC_50_ values of 2.8, 6.7, and 1.4 µM, respectively (Figure 2B). The results suggest that 11Cha1 displays an inhibitory activity on the inflammasome activation.

### 2.3. 11Cha1 Inhibits the Activation of NF-ĸB but not MAPKs

**Figure 3:**
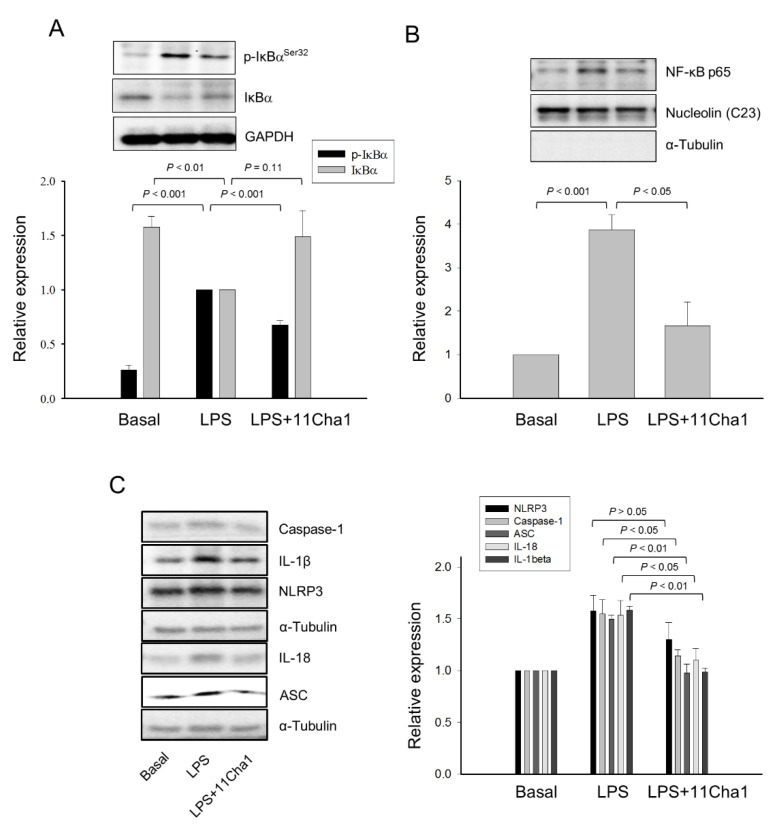
Effect of 11Cha1 on LPS-induced IĸBα phosphorylation, p65 NF-ĸB nuclear translocation and several protein expressions. THP-1 cells were seeded and differentiated by 50 nM PMA for 48 h. Then, the cells were pre-treated in the absence or presence of 30 µM 11Cha1 for 30 min and then treated with 0.3 µg/mL LPS for 30 min (**A**,**B**) or 2 h (**C**). The cells were harvested for the detection of p-IkBα and IkBα (**A**), nuclear translocation of p65 NF-ĸB (**B**), or several protein expressions (**C**) using Western blot analysis. Data are expressed as mean ± SEM of at least three independent determinations.

NF-ĸB plays a key role in the priming step of NLRP3 inflammasome activation through inducing the transcriptional expression of inflammasome components in response to LPS/toll-like receptor 4 (TLR4) signaling activation [8,9,10,21,23]. We performed an experiment that after the treatment of cells with LPS for 8 h, the medium was replaced with LPS-free fresh medium for a further 16-h incubation to allow IL-6 production. The data showed that 11Cha1 significantly inhibited the IL-6 release in LPS-free condition, suggesting the inhibition of intracellular but not extracellular signaling to 11Cha1 action (Supplementary Materials Figure S2). The effect of 11Cha1 on the regulation of NF-ĸB activation was determined. NF-ĸB activation needs the phosphorylation and proteolytic degradation of the inhibitory subunit IĸB-α. The data demonstrated that a 30 min-exposure to LPS induced a profound increase of IĸB-α phosphorylation associated with its proteolytic degradation. These effects were significantly inhibited by 11Cha1 (Figure 3A). NF-ĸB activation is connected to the nuclear translocation of p65 component of the complex. The nuclear extracts were prepared showing that LPS induced a dramatic increase of nuclear translocation of NF-ĸB p65 subunit. The effect also was significantly inhibited by 11Cha1 (Figure 3B). Notably, LPS significantly increased the protein expression levels of NLRP3, pro-caspase-1, ASC, IL-18, and IL-1β. 11Cha1 resulted in a profound suppression on these proteins except for NLRP3, which showed an inhibitory trend (Figure 3C).

Moreover, IL-6 was reported to be one of the abundant cytokines induced by NF-ĸB-dependent activation pathway [24]. Our data showed that LPS resulted in a profound IL-6 release, which was inhibited by 11Cha1 in a concentration-dependent fashion with an IC_50_ of 5.16 ± 0.54 µM (Supplementary Materials Figure S3). Besides NF-ĸB, the MAPKs including ERK, JNK, and p38 MAPK in the priming process to regulate inflammatory responses have emerged recently [25]. However, 11Cha1 did not inhibit LPS-induced activation of MAPKs (Supplementary Materials Figure S4). Altogether, the data indicated that the activation of NF-ĸB but not MAPKs was predominantly responsible for the upregulation of the inflammasome components and confirmed that 11Cha1 inhibited LPS-induced priming effect on NLRP3 inflammasome components through the suppression of NF-ĸB activity.

### 2.4. 11Cha1 Inhibits NLRP3-Dependent ASC Oligomerization and Caspase-1 Activation

**Figure 4:**
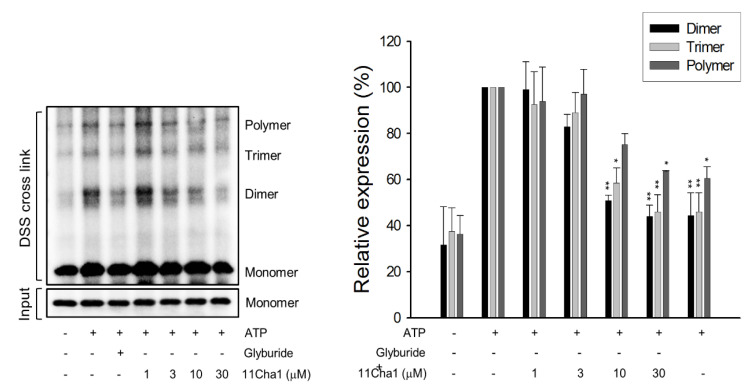
Effect of 11Cha1 on ATP-induced ASC dimerization and oligomerization. THP-1 cells were seeded and differentiated by 50 nM PMA for 48 h and then primed with 1 μg/mL LPS for 4 h. The cells were pre-treated with or without the indicated agent for 30 min (200 µM glyburide as the positive control) and then treated with 5 mM ATP for another 2 h. After treatment, the cells were harvested for the detection of the protein expressions of ASC dimerization and oligomerization. Data are expressed as mean ± SEM of at least three independent determinations. One-way ANOVA by Newman Keuls post hoc test was used. * *P* < 0.05 and ** *P* < 0.01 compared with ATP-treated control. The analyses of *P* (ANOVA) indicated *P* < 0.01 in all tests.

**Figure 5:**
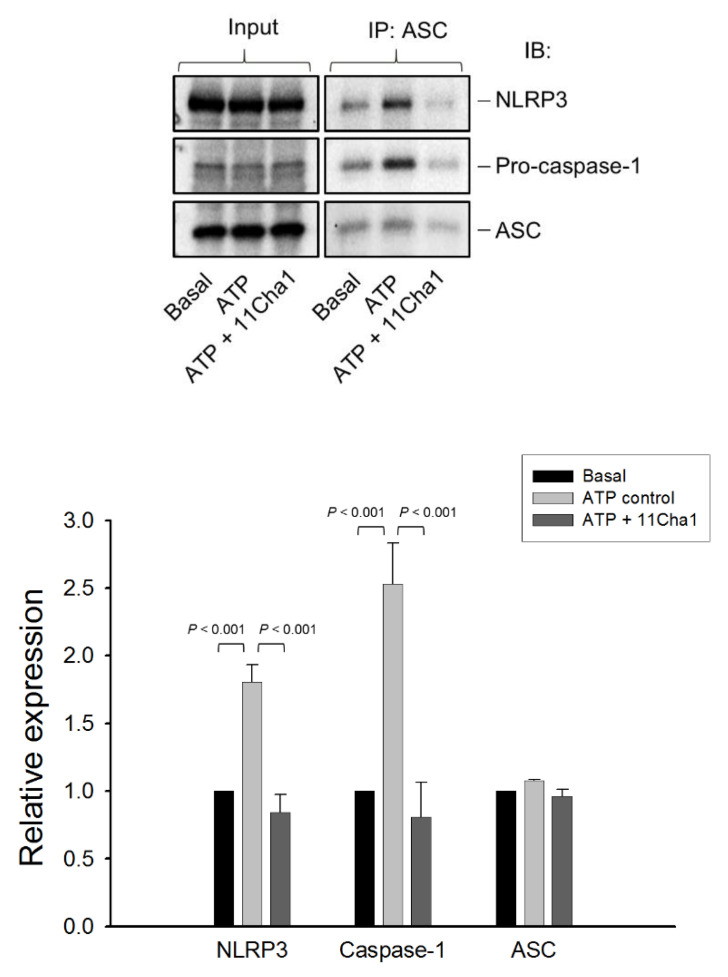
Effect of 11Cha1 on ATP-induced interaction between NLRP3, pro-caspase-1, and ASC. THP-1 cells were seeded and differentiated by 50 nM PMA for 48 h and then primed with 1 μg/mL LPS for 4 h. The cells were pre-treated with or without 11Cha1 (30 µM) for 30 min and then treated with 5 mM ATP for another 2 h. After treatment, immunoprecipitation and immunoblotting experiments were performed. Data are expressed as mean ± SEM of four independent determinations. One-way ANOVA by Newman Keuls post hoc test was used. The analyses of *P* (ANOVA) indicated *P* < 0.001 in all tests.

The NLRP3 inflammasome consisted of sensor molecule NLRP3, adaptor protein ASC, and pro-caspase-1. The NLRP3 harbored a pyrin domain (PYD) and ASC contained PYD and caspase activation and recruitment domain (CARD). Upon activation, the NLRP3 molecule interacted with ASC through PYD, while ASC recruited pro-caspase-1 via CARD domain to form NLRP3-ASC-pro-caspase-1 complex [9,26]. Both ASC dimerization and oligomerization were considered to be direct evidence for the inflammasome activation. The data demonstrated that ATP application in LPS-primed cells significantly induced the formation of ASC dimers, trimers, and oligomers, in which the dimer is the most apparent form. These effects were inhibited by glyburide (an NLRP3 inflammasome inhibitor) and 11Cha1 in a concentration-dependent manner (Figure 4). Co-immunoprecipitation and immunoblot analysis demonstrated that ATP induced increased levels of NLRP3 and pro-caspase-1 protein expressions in the assembly of NLRP3 inflammasome in LPS primed cells. The reaction was completely abolished by 11Cha1 (Figure 5).

### 2.5. 11Cha1 Suppresses ATP-Induced K^+^ Efflux and Pyroptosis in LPS-Primed Macrophages

**Figure 6:**
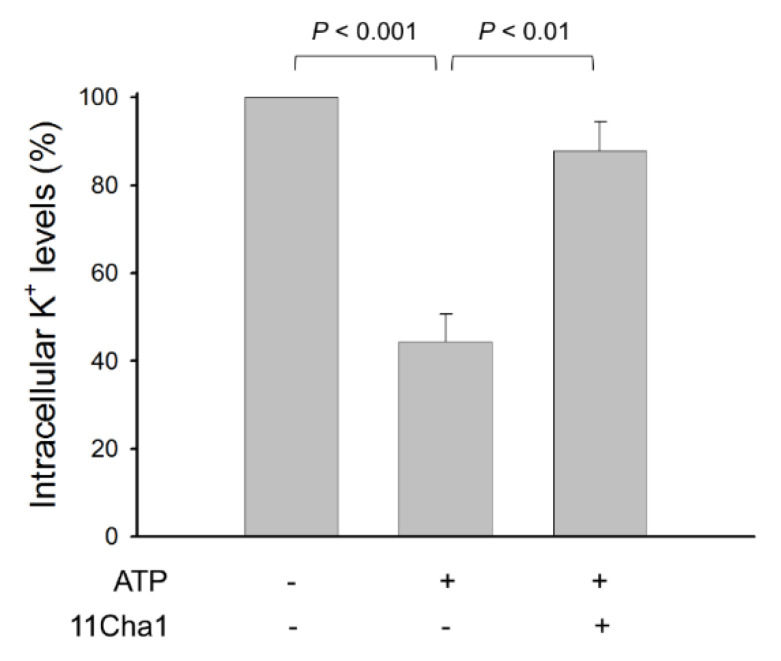
Effect of 11Cha1 on ATP-induced K^+^ outflow from cells. THP-1 cells were seeded and differentiated by 50 nM PMA for 48 h and then primed with 1 μg/mL LPS for 4 h. The cells were pre-treated with or without 11Cha1 (30 µM) for 30 min and then treated with 5 mM ATP for another 30 min. After treatment, intracellular potassium content was determined using Potassium Assay Kit (MyBioSource, San Diego, CA, USA) according to manufacturer’s protocols. Data are expressed as mean ± SEM of five independent determinations. One-way ANOVA by Newman Keuls post hoc test is used. The analyses of *P* (AVOVA) indicates *P* < 0.001.

The NLRP3 inflammasome is stimulated by multiple cellular events, including ionic flux, oxidative stress, mitochondrial dysfunction, and lysosomal damage [27]. Notably, although various stimuli have been identified, the common cellular activity elicited by all NLRP3 inflammasome activators is suggested to be the cell membrane permeability of K^+^ and Na^+^, in particular the decrease of intracellular K^+^ levels [9,21,27,28]. Our study showed that ATP, a P2X_7_ purinoceptor agonist, induced a dramatical K^+^ efflux in LPS-primed cells. However, this effect was substantially diminished by 11Cha1 (Figure 6), further verifying the anti-NLRP3 inflammasome activity of 11Cha1.

**Figure 7:**
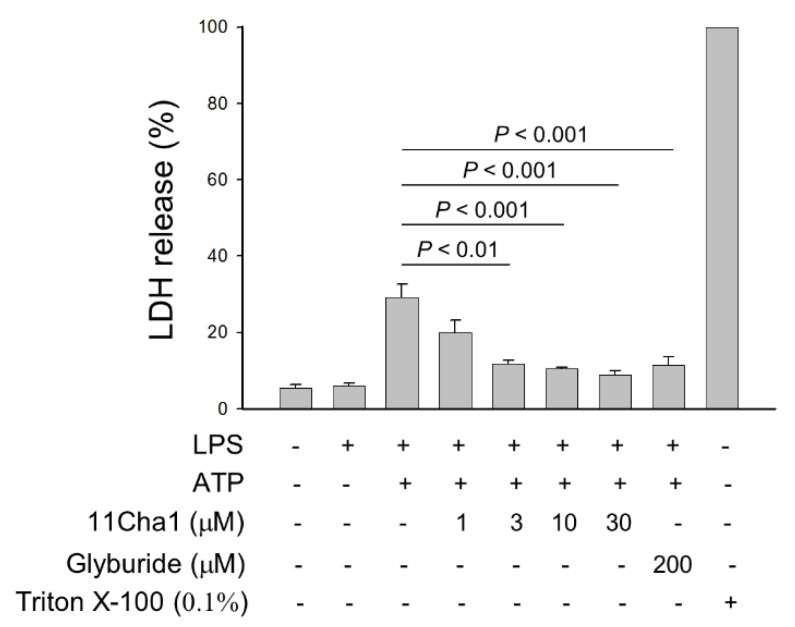
Effect of 11Cha1 on ATP-induced pyroptosis reaction. THP-1 cells were seeded and differentiated by 50 nM PMA for 48 h and then primed with 1 μg/mL LPS for 4 h. The cells were pre-treated with or without the indicated agent for 30 min and then treated with 5 mM ATP for another 2 h. After the treatment, the LDH levels in medium was determined using LDH assay kit. Data are expressed as mean ± SEM of five independent determinations.

Pyroptosis, an inflammatory form of programmed cell death, was mediated by caspase-1, 4, and -5 after the activation of inflammasomes, including NLRP1, NLRP3, AIM2, NLRC4, and Pyrin [29]. Among them, NLRP3 is the most important NOD-like receptor protein in recognizing microbial and other danger signals to produce a sterile inflammatory reaction. Pyroptosis may take place in immune cells, keratinocytes, and epithelial cells. Pyroptosis is often examined using an assessment to determine the release of lactate dehydrogenase (LDH) into the culture media. Therefore, the pyroptosis reaction was examined in the present study using enzymatic assays for LDH detection in commercially available kit. As demonstrated in Figure 7, 11Cha1 inhibited LPS/ATP-induced LDH release in a concentration-dependent fashion, further substantiating the anti-NLRP3 inflammasome activity of 11Cha1.

## 3. Discussion

A variety of stimuli were capable of inducing inflammasome activation in response to cellular stress and sensing microbial molecules. Dysregulated inflammasome activity, in particular NLRP3 inflammasome, was linked to several inflammatory diseases, such as diabetes, atherosclerosis, inflammatory bowel disease, multiple sclerosis, vitiligo, and gouty arthritis [30,31,32,33]. The study of small molecule inhibitors of the NLRP3 inflammasome was a potential approach to discover appropriate therapeutics for inflammatory disorders and to unveil the mechanism for future target therapy. The effects of several chalcone derivatives and flavonoids on inhibition of NLRP3 inflammasome activation were examined in this study. Chalcone belongs to the flavonoid family due to the reason that it acts as a precursor in the biosynthetic pathway of flavonoid compounds. In contrast to the chromone moiety in traditional flavonoid, chalcone is structurally characterized with a less hindered α, β-unsaturated ketone group. This group is able to function as a Michael acceptor, which induces the nucleophilic attack by the amino residue on intracellular target molecule protein triggered the signaling pathway. In the present study, 11Cha1 showed higher activity than the other derivatives in anti-inflammasome effects. A family of TLRs serve as primary sensors in responding to a variety of microbial components for producing innate immune responses. TLR signaling cascades lead to nuclear translocation and activation of NF-ĸB that regulates the expression of various inflammatory cytokine genes. NF-ĸB also is crucial in the priming process of NLRP3 inflammasome activation through triggering transcriptional expression of inflammasome components [8,9,10,21,23]. NF-ĸB activation needs the phosphorylation and degradation of the inhibitory protein, IĸB [34]. Our data showed that 11Cha1 substantially inhibited LPS-induced IĸBα phosphorylation and degradation, NF-ĸB nuclear translocation, upregulation of NLRP3 inflammasome components, and IL-6 production, suggesting that the suppression of NF-ĸB-dependent pathway contributed to 11Cha1-mediated inhibition of step 1 priming process in NLRP3 inflammasome activation.

Although NF-ĸB-dependent IL-1β and NLRP3 expression is considered the major priming effect, several studies have highlighted the events other than NF-ĸB-dependent transcription pathway. Song and the colleagues, by using knockin of NLRP3-S194A mutant in mice, have reported that JNK1-mediated NLRP3 phosphorylation at Ser194 during the priming step is crucial to introduce the oligomerization of NLRP3 [35]. Ghonime and colleagues focused on endotoxin priming within minutes that were independent of new mRNA and protein synthesis, showing that ERK inhibition and small interfering RNA-mediated ERK1 knockdown significantly suppressed the priming effect. Their study suggested that ERK1-involved posttranslational regulation dominates the priming process [36]. In addition to JNK and ERK, p38 is also a widely studied MAPK in regulating inflammatory reaction [37,38]. Recently, the p38α-MAPK activated protein kinase 2 (MK2) complex in mediating inflammasome priming was examined. The use of CDD-450, a unique inhibitor selectively blocking p38α activation of the proinflammatory kinase MK2, demonstrated little effect on NLRP3 expression but reduced IL-1β expression through stimulating the degradation of IL-1β mRNA in both bone marrow macrophages and in vivo disease model [39]. The study suggests that, besides NF-ĸB-dependent translational pathway and inflammasome-mediated posttranslational reaction, IL-1β can be regulated posttranscriptionally by p38α-MK2. Our data demonstrated that 11Cha1 inhibited LPS-induced upregulation of NLRP3 inflammasome components, suggesting the suppression occurred predominantly at LPS-mediated transcriptional pathways. Besides, the fail of 11Cha1 on modifying MAPKs activities revealed that 11Cha1 did not regulate the posttranslational pathways during the priming process of NLRP3 inflammasome activation. Another factor in regulating NLRP3 inflammasome activation is reactive oxygen species (ROS). ROS has been suggested to function as a triggering factor in NLRP3 inflammasome activation [40]; however, some reports document that ROS production is dispensable for NLRP3 inflammasome activation [41,42]. Our data demonstrated that 11Cha1 at the concentrations used for inhibiting NLRP3 inflammasome activation did not affect LPS/ATP-mediated increase of ROS production (Supplementary Materials Figure S5), indicating ROS-independent mechanism to 11Cha1 action.

Several ion fluxes, including K^+^ efflux, Na^+^ influx, Cl^-^ efflux, and Ca^2+^ mobilization, were identified as crucial signaling in NLRP3 inflammasome activation, in which a decrease of intracellular K^+^ concentration is suggested as a common step [9,21,27,28]. NEK7, a member of the family of mammalian NIMA-related kinases (NEK proteins), was identified recently as an NLRP3-binding component that also works downstream of K^+^ efflux during NLRP3 inflammasome activation [43]. Notably, a K^+^ efflux-independent pathway for NLRP3 inflammasome activation was reported recently. Groß and colleagues demonstrated that imiquimod (an immune response modifier by activating TLR7) and the related molecule CL097 suppress the quinone oxidoreductases NQO2 and mitochondrial Complex I, leading to reactive oxygen species production and thiol oxidation, which cause NLRP3 inflammasome activation through NEK7 without K^+^ efflux [44]. In this regard, our data demonstrated that ATP induced a dramatic K^+^ efflux and NLRP3 inflammasome activation in LPS-primed cells. These effects were substantially inhibited by 11Cha1, suggesting an essential role of K^+^ efflux in NLRP3 inflammasome activation and confirming the inhibitory effect of 11Cha1 on K^+^ efflux. Furthermore, another experiment using poly(dA:dT), a repetitive synthetic dsDNA sequence of poly(dA-dT)·poly(dT-dA), to induce absent in melanoma 2 (AIM2, a cytosolic double-stranded DNA sensor in recognizing double-stranded DNA of microbial or host origin) in inflammasome activation [45]. The data showed that 11Cha1 also significantly attenuated poly(dA:dT)-induced IL-1β release, suggesting the inhibitory effect of 11Cha1 on AIM2 inflammasome (Supplementary Materials Figure S6). Our data of 11Cha1-mediated inhibition on both NLRP3 and AIM2 inflammasome suggest its block on a common upstream pathway.

In conclusion, the structure-activity relationship of chalcone derivatives in anti-inflammasome activities and the mechanisms were dissected through which 11Cha1 can inhibit both priming and activation steps. 11Cha1 inhibited the priming process of NF-ĸB activation and subsequently suppressed the up-regulation of NLRP3 inflammasome components including NLRP3, ASC, pro-caspase-1, pro-IL-18, and pro-IL-1β. Next, 11Cha1 inhibited ATP-mediated K^+^ efflux and blocked the assembly and activation of the NLRP3 inflammasome, which inhibited caspase-1 activation and proteolytic cleavage, maturation, and secretion of IL-1β and IL-18. Since inflammasomes are responsible for the activation of inflammatory responses through triggering proteolytic cleavage, maturation and secretion of several major inflammatory cytokines. Dysregulation of inflammasome activation may cause a variety of major diseases, such as autoimmune, metabolic, and neurodegenerative diseases and cancer. Our results also suggest the significance to human health by the fact that vegetables, fruits, teas, and medicinal plants are rich sources of chalcones and flavonoids that have been linked to reducing the risk of inflammation and a variety of major chronic diseases.

## 4. Materials and Methods

### 4.1. Materials

RPMI 1640 medium, PSA Solution (100 U/mL penicillin, 0.1 mM Streptomycin, 250 nM Amphotericin B) and fetal bovine serum (FBS), sodium pyruvate were obtained from GIBCO/BRL Life Technologies (Grand Island, NY, USA). Antibodies to α-tubulin, NF-κB p65, and HRP-conjugated anti-mouse and anti-rabbit IgG were obtained from Santa Cruz Biotechnology, Inc. (Santa Cruz, CA, USA). Antibodies to Caspase-1, ASC, NLRP3, p-p44/42 MAPK (Erk1/2)^Thr202/Tyr204^, p-p38 MAPK^Thr180/Tyr182^, p-IκB^Ser32^, IκB, IL-1β, nucleolin (C23), and GAPDH were from Cell Signaling Technologies (Boston, MA, USA). Antibodies to IL-18 and p-JNK1/2/3^(Y185/Y185/Y223)^ were from ABCam (Cambridge, MA, USA). Potassium Assay Kit was from MyBioSource (San Diego, CA, USA). LPS, ATP, trichloroacetic acid (TCA), PMA, acetone, D-glucose, NaHCO_3_, dithiothreitol, phenylmethylsulfonylfluoride (PMSF), MTT, leupeptin, NaF, NaVO_4_, disuccinimidyl suberate (DSS), and all other chemical compounds were obtained from Sigma-Aldrich (St. Louis, MO, USA). Human Caspase-1/ICE (DCA100), IL-6 (D6050), and IL-1β/IL-1F2 (DLB50) Immunoassay Kits were from R&D system (Minneapolis, MN, USA). Human IL-18 ELISA kit was from MBL (Nagoya, Japan). Bio-Red protein assay kit was from Bio-Red (Hercules, CA, USA). The synthesis and structure identification of chalcone derivatives are demonstrated in the Appendix A.

### 4.2. Cell Lines and Cell Culture

THP-1 human monocytic cell line was obtained from the Bioresources Collection and Research Center of the Food Industry Research and Development Institute (Hsinchu, Taiwan). Cells were cultured in RPMI 1640 medium containing 10% inactivated FBS, 100 U/mL penicillin, 0.1 mM Streptomycin, 250 nM Amphotericin B, 2.5 g/L glucose, and 1 mM sodium pyruvate. Cells were maintained in a humidified incubator at 37 °C in 5% CO_2_/95% air.

### 4.3. MTT Assays

Cells were differentiated in the presence of 50 nM PMA for 48 h to macrophage. The cells were treated with 0.3 μg/mL LPS in the absence or presence of 11Cha1 for 3 or 24 h. After the treatment, 0.5 mg/mL MTT (dissolved in PBS) was added for another 2 h. Finally, the formed formazan was dissolved in 0.1 mL DMSO for 5 min. The absorbance was read at a wavelength of 590 nm.

### 4.4. Cytokine Release

Cells were differentiated in the presence of 50 nM PMA for 48 h to macrophage. After the treatment, the cells were treated with or without 1 μg/mL LPS for 3 h. Then, the cells were treated with the indicated agent for 30 min and then treated with 5 mM ATP and the indicated agent for another 2 h. After the treatment, cytokine concentrations in the medium were quantified using ELISA kits according to the manufacturer’s protocols. Briefly, the resulting medium was added to the wells of microplate pre-coated with the monoclonal antibody specific for target cytokine. After a two-hour incubation at room temperature and washing, specific cytokine conjugate was added for further one-hour incubation. After washing, substrate solution was added with another 20-min incubation. Then, the stop solution was added and the color was developed for the determination of the optical density.

### 4.5. Western Blotting

After the treatment, the cells or the resulting medium were obtained. For cellular protein, the cells were lysed in 0.1 mL of lysis buffer (10 mM Tris-HCl pH 7.4, 150 mM NaCl, 1mM EGTA, 1% Triton X-100, 1 mM PMSF, 10 μg/mL leupeptin, 1 mM DTT, 50 mM sodium fluoride, and 1 mM sodium orthovanadate) for 30 min at 4 °C. After centrifugation, the supernatants were obtained and the protein concentrations were quantified. In other experiments, the protein in resulting medium was precipitated by 20% TCA for 30 min on ice. After centrifugation (12,000 rpm, 20 min), the pellets were washed twice by acetone. For Western blotting, proteins (30 μg) were separated by electrophoresis in a 10 or 14% polyacrylamide gel and were transferred to a PVDF membrane. After an hour incubation at room temperature in PBS/5% non-fat milk, the membrane was washed with PBS/0.1% Tween 20 for another 1 h and incubated in the presence of the indicated antibody overnight at 4 °C. After three washings with PBS/0.1% Tween 20, the membranes were incubated with anti-mouse or anti-rabbit IgG (dilute 1:8000) for 1 h at room temperature. After washings with PBS/0.1% Tween 20, the protein expressions were detected with an enhanced chemiluminescence detection kit (Amersham, Buckinghamshire, UK) and the membranes were scanned using a ChemiDoc^™^ MP Imaging System (BIO-RAD, Hercules, CA, USA).

### 4.6. ASC Oligomerization

After the treatment, the cells were suspended in 0.1 ml buffer A (20 mM HEPES pH 7.4, 10 mM KCl, 1.5 mM MgCl_2_, 1 mM EGTA, 1 mM EDTA, 1 mM PMSF, 10 μg/mL leupeptin, 1 mM sodium fluoride, and 1 mM sodium orthovanadate) and lysed by shearing 30 times using a 27-gauge needle. The lysate (20 µL) was obtained serving as loading input protein. The lysates were centrifuged at 300× *g* for 8 min at 4 °C. The supernatants were collected and diluted with equal volume of CHAPS lysis buffer and centrifuged (2600× *g*, 8 min, 4 °C). The resulting pellets were re-suspended in 20 µL lysis buffer containing 4 mM of DSS. The samples were incubated at room temperature for 30 min to cross-link proteins and then mixed with sample buffer and boiled at 90 °C (5 min) for Western blotting.

### 4.7. Measurement of Intracellular Potassium Content

After the treatment, the cells were suspended in 0.3 mL ddH_2_O and sonicated in ice water bath (power: 300 W for 3 seconds, interval for 30 seconds, repeat for 5 times). After sonication, the protein concentration of homogenate liquid was quantified and the intracellular potassium content was determined using Potassium Assay Kit (MyBioSource, San Diego, CA, USA) according to manufacturer’s protocols. Briefly, the commercial protein precipitant was added into 20 μL of homogenate liquid. After centrifugation, the supernatant was obtained and mixed with working solution for 5 min. The optical density was determined at 450 nm. Data were normalized based on the protein concentration of the homogenate liquid.

### 4.8. Immunoprecipitation Assay

After the treatment, the cells were collected and centrifuged (2000 rpm, 10 min, 4 °C). The cell pellet was resuspended in IP lysis buffer (0.5 mL, 50 mM HEPES, pH 7.4, 150 mM NaCl, 10% Glycerol, 2 mM EDTA, 0.5% Triton X-100, 1 mM PMSF, 10 μg/mL leupeptin, 1 mM DTT, 50 mM sodium fluoride, and 1 mM sodium orthovanadate) for 30 min on ice and then centrifuged at 12,000 rpm for 20 min at 4 °C. The protein concentration of supernatant was determined. The supernatant (500 μg) was immune-precipitated with 1 μg antibody against ASC at 4 °C overnight. A/G magnetic beads were added to each sample and incubated at 4 °C overnight. After washings of the beads with IP lysis buffer, the beads-bound proteins were mixed with sample buffer and boiled at 90 °C for 5 min for immunoblotting.

### 4.9. Lactate Dehydrogenase (LDH) Release

Cells were differentiated in the presence of 50 nM PMA for 48 h to macrophage. After the treatment, the cells were treated with or without 1 μg/mL LPS for 4 h. Then, the cells were treated with the indicated agent for 30 min and then treated with 5 mM ATP for another 2 h. After the treatment, LDH concentrations in the medium were quantified using ELISA kits according to the manufacturer’s protocols method (Promega, Madison, WI, USA). Briefly, the resulting medium was added to the fresh 96-well plate with CytoTox 96 reagent. After a 10-min incubation at room temperature, the stop solution was added and the color was developed for the determination of the optical density. Total release of LDH was measured by incubating cells with lysis buffer (0.1% Triton X-100) for 45 min.

### 4.10. Data Analysis

Data are presented as mean±SEM for the indicated number of independent experiments. One-way ANOVA followed by a Newman Keuls post hoc test was applied. *P*-values less than 0.05 were considered statistically significant.

## Figures and Tables

**Table 1:** IC_50_ values of the inhibition by chalcone and flavonoid derivatives against LPS-primed ATP-induced pro-caspase-1 processing in differentiated THP-1 cells.

	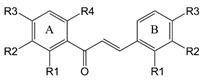			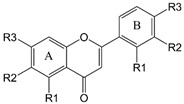		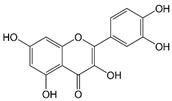		
	Chalcones			Flavonoids		Quercetin		
	A ring				B ring			
Chalcones	−R1	−R2	−R3	−R4	−R1	−R2	−R3	IC_50_ (μM)
11Cha1	−OH	−H	−OCH_3_	−OCH_3_	−H	−H	−COOH	1.5
11Cha2	−OH	−H	−OMOM ^a^	−OMOM ^a^	−H	−H	−COOH	1.7
11Cha3	−H	−H	−OCH_3_	−H	−H	−Br	−OH	1.9
11Cha4	−H	−H	−OCH_3_	−H	−H	−H	−I	>30
11Cha5	−H	−H	−OH	−H	−H	−H	−OCH_2_COOCH_3_	3.8
11Cha6	−OMOM ^a^	−OCH_3_	−OMOM ^a^	−OH	−H	−H	−OBn ^b^	>30
11Cha7	−H	−H	−OBn ^b^	−H	−H	−H	−OH	2.3
11Cha8	−H	−H	−H	−H	−OCH_2_COOCH_2_CH_3_	−H	−H	2.4
11Cha9	−OBn ^b^	−I	−OMOM ^a^	−OH	−H	−H	−OBn ^b^	>30
11Cha10	−OBn ^b^	−OCH_3_	−OH	−OH	−H	−H	−OBn ^b^	>30
11Cha11	−OBn ^b^	−OCD_3_	−OH	−OH	−H	−H	−OBn ^b^	>30
Flavonoids	−R1	−R2	−R3	−R1	−R2	−R3	Release (%)	IC_50_ (μM)
Control							100±0	
Apigenin	−OH	−H	−OH	−H	−H	−OH	89.5±18.1^c^	>30
Luteolin	−OH	−H	−OH	−H	−OH	−OH	94.2±12.4 ^c^	>30
(±)−Hesperetin	−OH	−H	−OH	−H	−OH	−OCH_3_	58.1±11.6 ^c^	>30
Naringenin	−OH	−H	−OH	−H	−H	−OH	59.0±9.3 ^c^	>30
Hispidulin	−OH	−OCH_3_	−OH	−H	−H	−OH	79.6±19.1^c^	>30
Quercetin							81.3±15.6 ^c^	>30

^a^ −OCH_2_OCH_3_, ^b^ −OCH_2_C_6_H_5_, ^c^ Caspase-1 p20 release (%) in the presence of 30 μM flavonoid.

**Figure A1:**
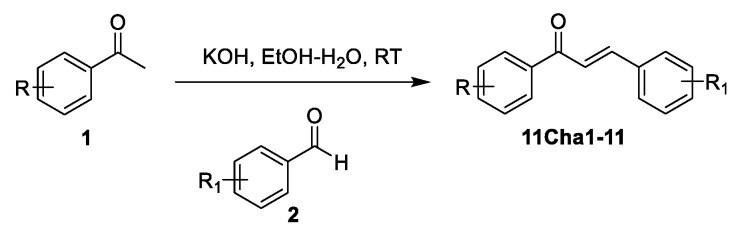
General procedure for the synthesis of compound 11Cha1–11.

## Supplementary Materials

The following are available online. Figure S1: Effect of 11Cha1 on cytotoxic effect, Figure S2: Effect of 11Cha1 on intracellular signaling-mediated IL-6 release reaction, Figure S3: Effect of 11Cha1 on IL-6 release reaction, Figure S4: Effect of 11Cha1 on LPS-induced MAPK phosphorylation, Figure S5: Effect of 11Cha1 and NAC on inhibition of ROS production, Figure S6: Effect of 11Cha1 on poly (dA:dT)-induced IL-1b release.

## Abbreviations

ASC	apoptosis-associated speck-like protein containing a CARD
ATP	adenosine triphosphate
CARD	caspase activation and recruitment domain
DAMPs	damage-associated molecular patterns
DSS	disuccinimidyl suberate
FBS	fetal bovine serum
JAK	Janus kinase
JNK	c-Jun N-terminal kinase
LPS	lipopolysaccharide
MAPK	Mitogen-activated protein kinase
MK2	MAPK activated protein kinase 2
mTOR	mammalian target of rapamycin
MSU	monosodium urate
NF-ĸB	nuclear factor-ĸB
NLRP3	NOD-, LRR- and pyrin domain-containing protein 3
PAMPs	pathogen-associated molecular patterns
PI3Ks	phosphoinositide 3-kinases
PMA	phorbol 12-myristate 13-acetate
PMSF	phenylmethylsulfonylfluoride
PYD	pyrin domain
STAT	signal transducers and activators of transcription
TCA	trichloroacetic acid
TLR4	toll-like receptor 4

## Appendix A

To a mixture of acetophenone 1 (1.57 mmol) and benzyloxybenzaldehyde 2 (1.73 mmol) in EtOH (4 mL) was added a solution of KOH (1.09 g) in EtOH-H_2_O (2 mL:1 mL) dropwise by syringe. The resulting solution was cooled to 0 °C and stirred for 3 h under N_2_. The reaction was then warmed to RT overnight. The reaction mixture was poured into ice water, acidified to pH 3–4 with 1N HCl_(aq)_ and then extracted with EtOAc (30 mL × 3). The combined organic layer was dried over Na_2_SO_4_, filtered, and the solvent removed in vacuo. The residue was purified by silica gel chromatography (EtOAc:*n*-hexane = 1:6) to give 11Cha1-11.

4-((*E*)-3-(2-Hydroxy-4,6-dimethoxyphenyl)-3-oxoprop-1-enyl)benzoic acid (11Cha1). ^1^H NMR (CDCl_3_, 300 MHz) δ 13.20 (1H, s), 7.98 (2H, d, *J* = 6.4 Hz), 7.84 (1H, s), 7.83 (1H, d, *J* = 15.0 Hz), 7.65 (1H, d, *J* = 15.0 Hz), 6.15 (2H, d, *J* = 6.4 Hz), 3.90 (3H, s), 3.82 (3H, s).

4-((*E*)-3-(2-Hydroxy-4,6-bis(methoxymethoxy)phenyl-3-oxoprop-1-enyl)benzoic acid (11Cha2). ^1^H NMR (CDCl_3_, 300 MHz) δ 13.16 (1H, s), 7.97 (2H, d, *J* = 8.4 Hz), 7.81 (2H, d, *J* = 8.4 Hz), 7.63 (1H, d, *J* = 15.0 Hz), 7.59 (1H, d, *J* = 15.0 Hz), 6.29 (1H, d, *J* = 2.2 Hz), 6.26 (1H, d, *J* = 2.2 Hz), 5.27 (2H, s), 5.22 (2H, s), 3.40 (3H, s), 3.37 (3H, s).

(E)-1-(4-Methoxyphenyl)-3-(3-bromo-4-hydoxyphenyl)prop-2-en-1-one (11Cha3).

^1^H NMR (DMSO-*d*_6_, 300 MHz) δ 11.01 (1H, s), 8.15 (2H, d, *J* = 8.9 Hz), 8.13 (1H, s), 7.80 (1H, d, *J* = 15.5 Hz), 7.68 (1H, dd, *J* = 2.1, 8.9 Hz), 7.59 (1H, d, *J* = 15.5 Hz), 7.06 (2H, d, *J* = 8.9 Hz), 6.99 (1H, d, *J* = 8.4 Hz), 3.85(3H, s).

(E)-1-(4-Methoxyphenyl)-3-(4-iodophenyl)prop-2-en-1-one (11Cha4).

^1^H NMR (CD_3_OD, 300 MHz) δ 8.11 (2H, d, *J* = 9.0 Hz), 7.83 (1H, d, *J* = 15.5 Hz), 7.82 (2H, d, *J* = 8.7 Hz), 7.69 (1H, d, *J* = 15.5 Hz), 7.53 (2H, d, *J* = 8.7 Hz), 7.07 (2H, d, *J* = 9.0 Hz).

Ethyl 4-(4-Methoxyphenyl-3-oxoprop-1-enyl)phenoxy acetate (11Cha5).

^1^H NMR (CDCl_3_, 300 MHz) δ 8.03 (2H, d, *J* = 8.0 Hz), 7.77 (1H, d, *J* = 12 Hz), 7.60 (2H, d, *J* = 8 Hz), 7.66 (1H, m), 7.58 (2H, m), 7.44 (1H, m), 7.06 (1H, m), 4.95 (2H, s), 4.20 (2H, q, *J* = 7.1Hz), 1.22 (3H, t, *J* =7.1 Hz).

(*E*)-1-(2-Hydroxy-4,6-bis(methoxymethoxy)phenyl)-3-(4-carbloxyoxyphenyl)prop-2-en-1-one (11Cha6).

^1^H NMR (CDCl_3_, 600 MHz) δ 13.25 (1H, s), 7.91 (1H, d, *J* = 15.5 Hz), 7.81 (1H, d, *J* = 15.5 Hz), 7.60 (2H, d, *J* = 8.7 Hz), 7.42 (2H, d, *J* = 7.2 Hz), 7.37 (2H, t, *J* = 7.2 Hz), 7.32 (1H, t, *J* = 7.2 Hz), 6.99 (2H, d, *J* = 8.7 Hz), 6.52 (1H, s), 5.25 (2H, s), 5.19 (2H, s), 5.10 (2H, s), 3.81 (3H, s), 3.50 (3H, s), 3.47 (3H, s)

(E)-1-(4-Benzyloxyphenyl)-3-(4-hydroxyphenyl)prop-2-en-1-one (11Cha7).

^1^H NMR (Acetone-*d*_6_, 300 MHz) δ 8.89 (1H, s), 8.14 (2H, d, *J* = 8.7 Hz), 7.71 (3H, m), 7.51 (2H, d, *J* = 8.1 Hz), 7.40 (3H, m), 7.15 (2H, d, *J* = 8.1 Hz), 6.92 (2H, d, *J* = 8.7Hz), 5.25 (2H, s).

Ethyl 2-(Phenyl-3-oxoprop-1-enyl)phenoxy acetate (11Cha8). ^1^H NMR (CDCl_3_, 300 MHz) δ 8.14 (2H, dd, *J* = 1.8, 7.1 Hz), 8.13 (2H, s), 7.93 (1H, dd, *J* = 1.8, 7.8 Hz), 7.66 (1H, m), 7.58 (2H, m), 7.44 (1H, m), 7.06 (1H, m), 4.95 (2H, s), 4.20 (2H, q, *J* = 7.1Hz), 1.22 (3H, t, *J* =7.1 Hz).

(E)-1-(2-(benzyloxy)-6-hydroxy-3-iodo-4-(methoxymethoxy)phenyl)-3-(4-(benzyloxy)phenyl)prop-2-en-1-one (11Cha 9).

1H-NMR (DMSO-*d*_6_, 300 MHz) δ 13.55 (1H, s), 7.84 (2H, m), 7.50 (4H, m), 7.42 (2H, m), 7.36 (3H, m), 7.26 (3H, m), 6.96 (2H, d, *J* = 8.8 Hz), 6.59 (1H, s), 5.41 (2H, s), 5.20 (2H, s), 4.91 (2H, s),3.51 (3H, s).

(E)-1-(2-Benzyloxy-4,6-dihydroxy-3-methoxyphenyl)-3-(4-benzyloxyphenyl)prop-2-en-1-one (11Cha10). ^1^H NMR (DMSO-d_6_, 300 MHz) δ 12.92 (1H, s), 10.52 (1H, s), 7.59 (2H, s), 7.48–7.45 (2H, m), 7.44–7.37 (5H, m), 7.32–7.29 (5H, m), 6.94 (2H, d, J = 8.8 Hz), 6.21 (1H, s), 5.16 (2H, s), 5.06 (2H, s), 3.77 (3H, s).

(E)-1-(2-Benzyloxy-4,6-dihydroxy-3-[^2^H_3_]methoxyphenyl)-3-(4-benzyloxyphenyl)prop-2-en-1-one (11Cha11).

^1^H NMR (DMSO-*d*_6_, 300 MHz) δ 12.95 (1H, s), 10.54 (1H, s), 7.59 (2H, s), 7.48–7.45 (2H, m), 7.44–7.36 (5H, m), 7.33–7.29 (5H, m), 6.94 (2H, d, *J* = 8.8 Hz), 6.22 (1H, s), 5.16 (2H, s), 5.06 (2H, s).
