# Supplementary figures and images for: Chalcones Display Anti-NLRP3 Inflammasome Activity in Macrophages through Inhibition of Both Priming and Activation Steps—Structure-Activity-Relationship and Mechanism Studies
Source: Molecules. 2020 Dec 16;25(24):5960. doi: 10.3390/molecules25245960 (PMC7767297; doi:10.3390/molecules25245960)

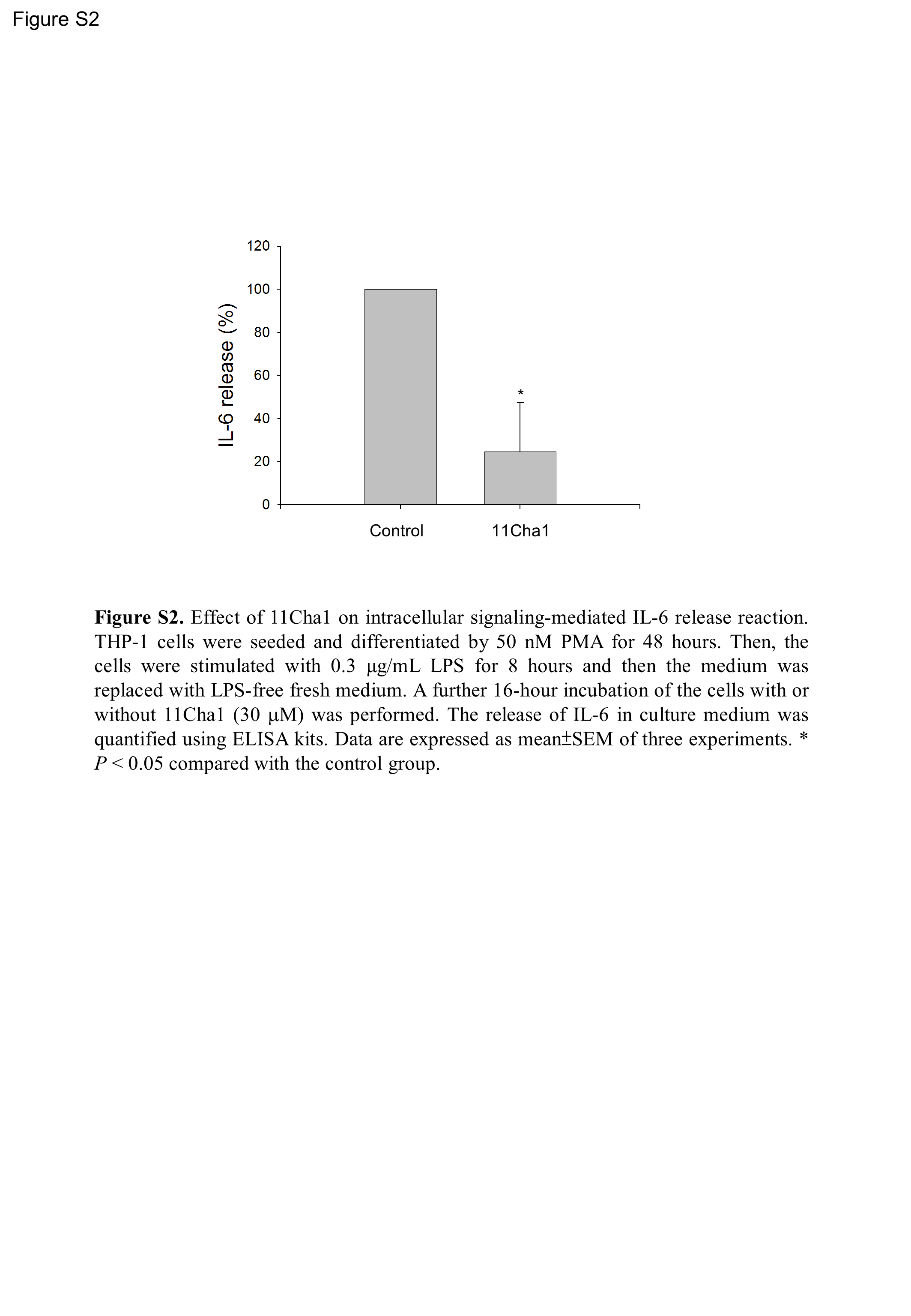

Supplement: Supplementary file 1 [file molecules-25-05960-s001.zip › Figure S2.tif]

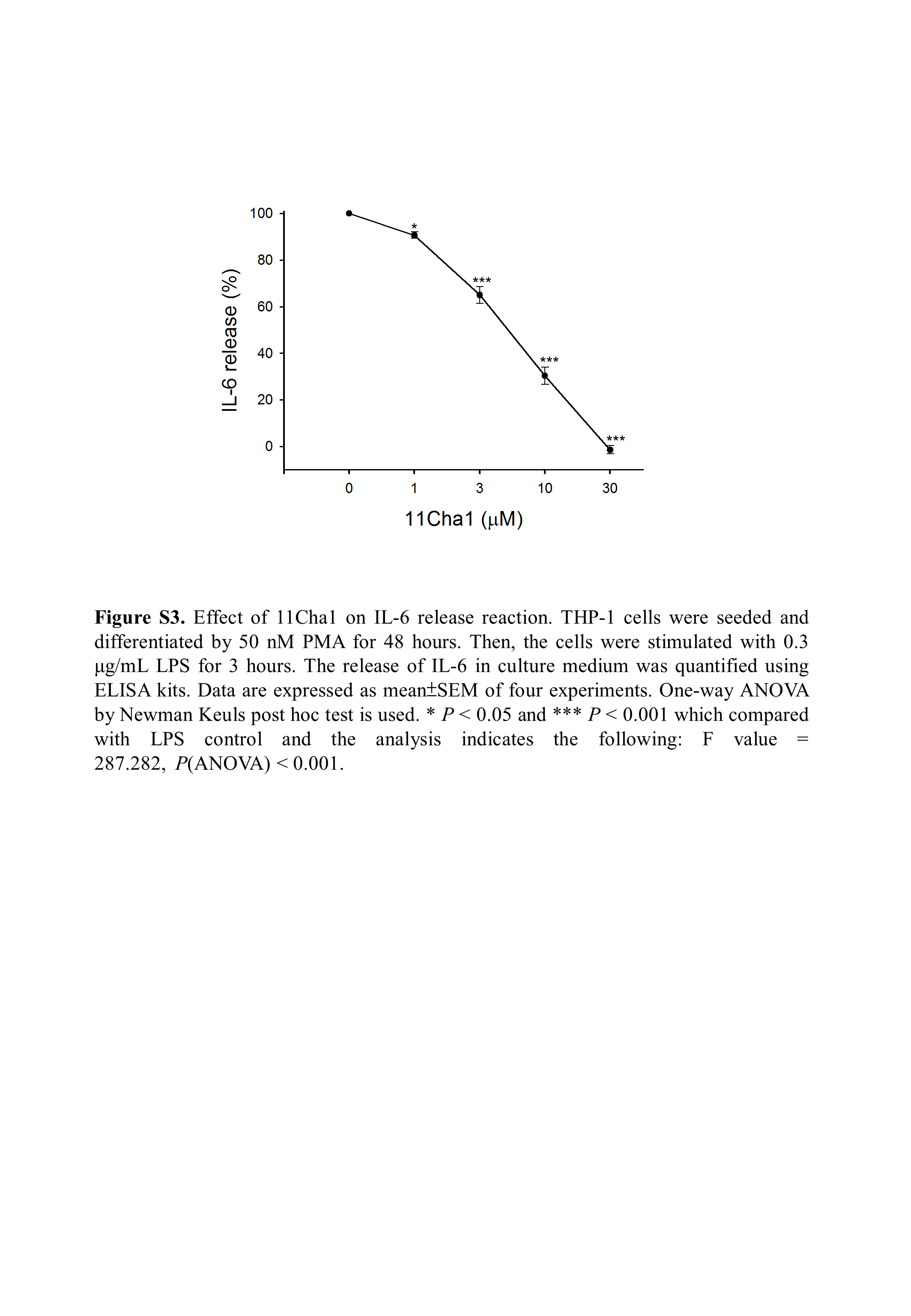

Supplement: Supplementary file 1 [file molecules-25-05960-s001.zip › Figure S3.tif]

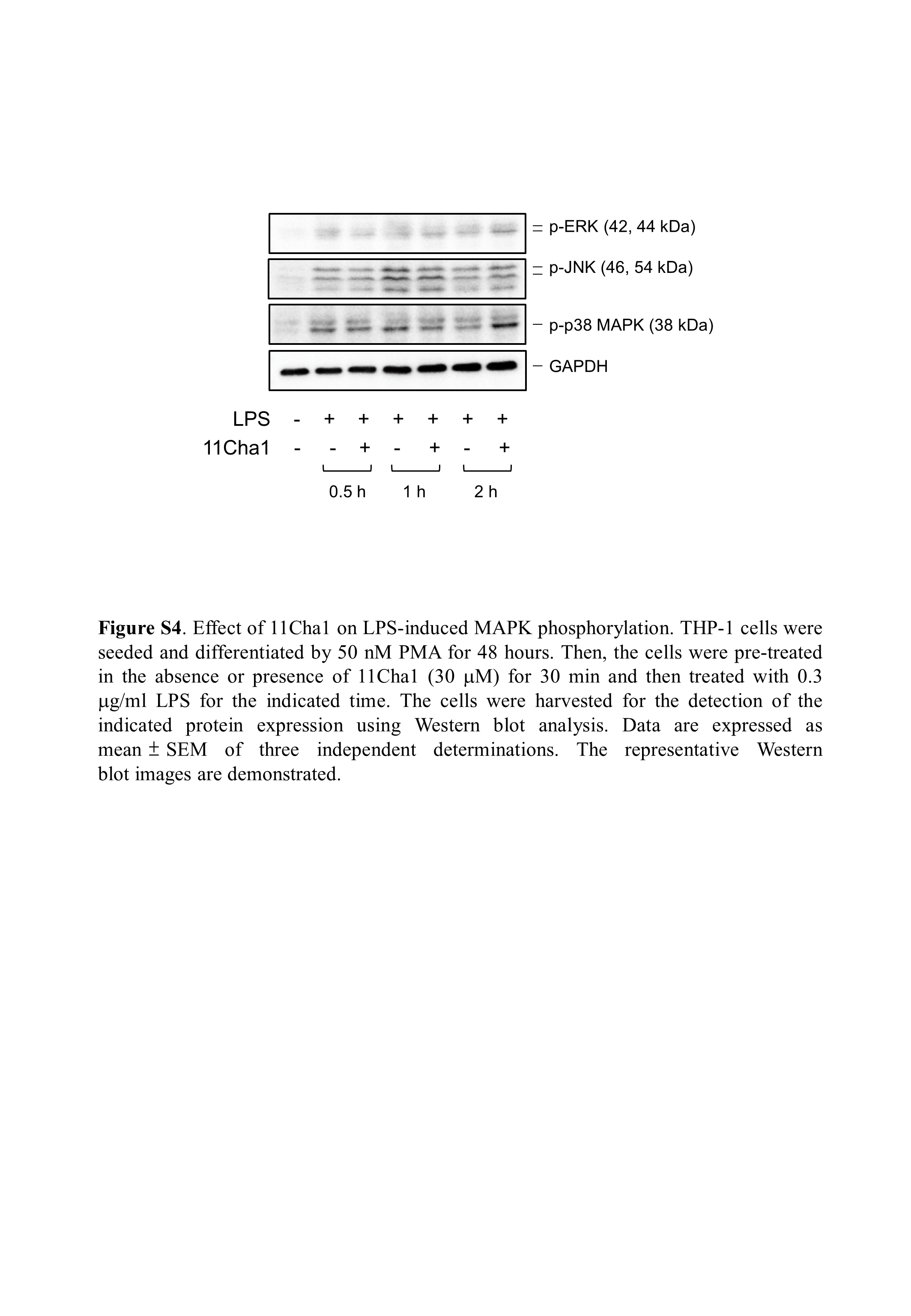

Supplement: Supplementary file 1 [file molecules-25-05960-s001.zip › Figure S4.tif]

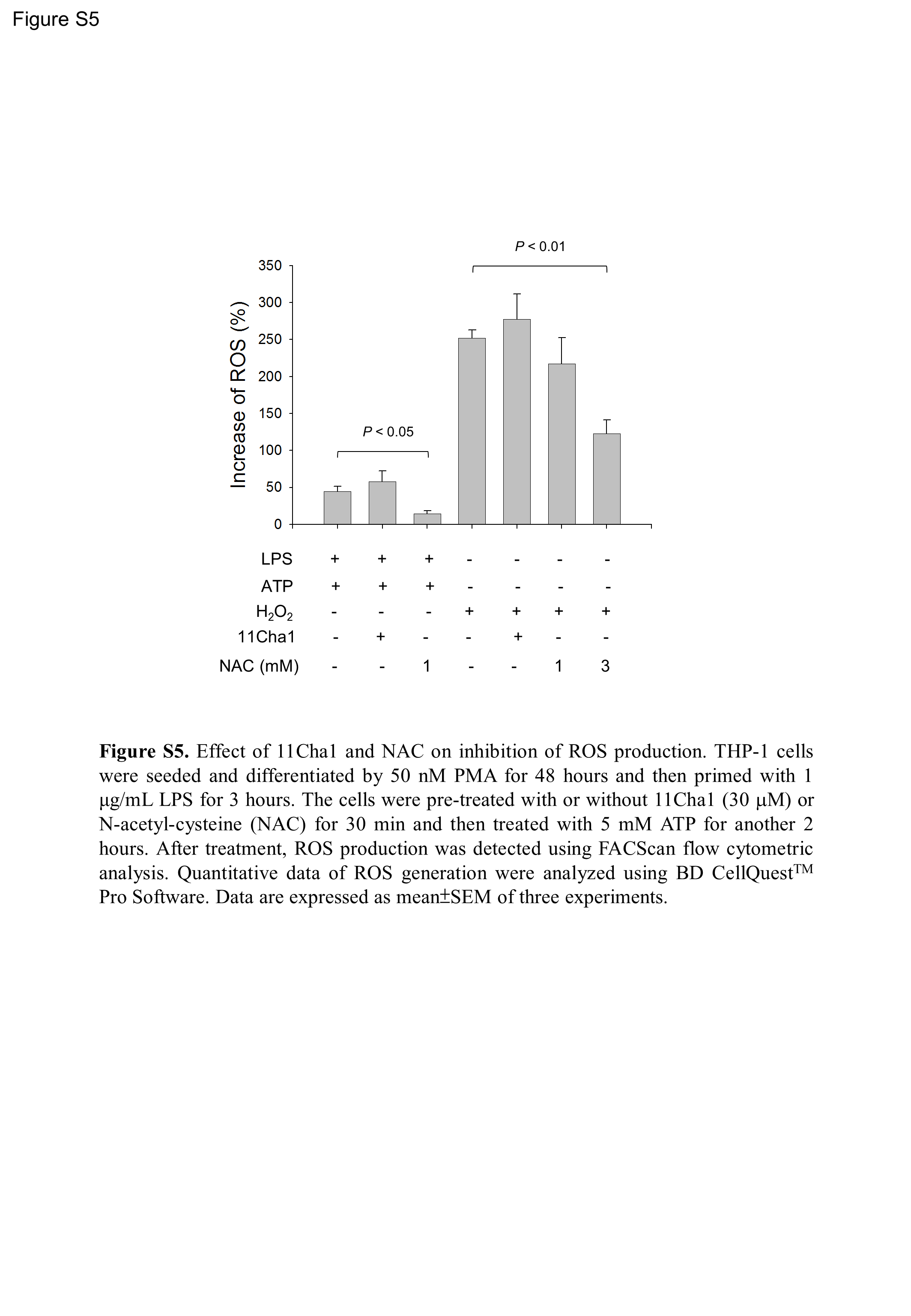

Supplement: Supplementary file 1 [file molecules-25-05960-s001.zip › Figure S5.tif]

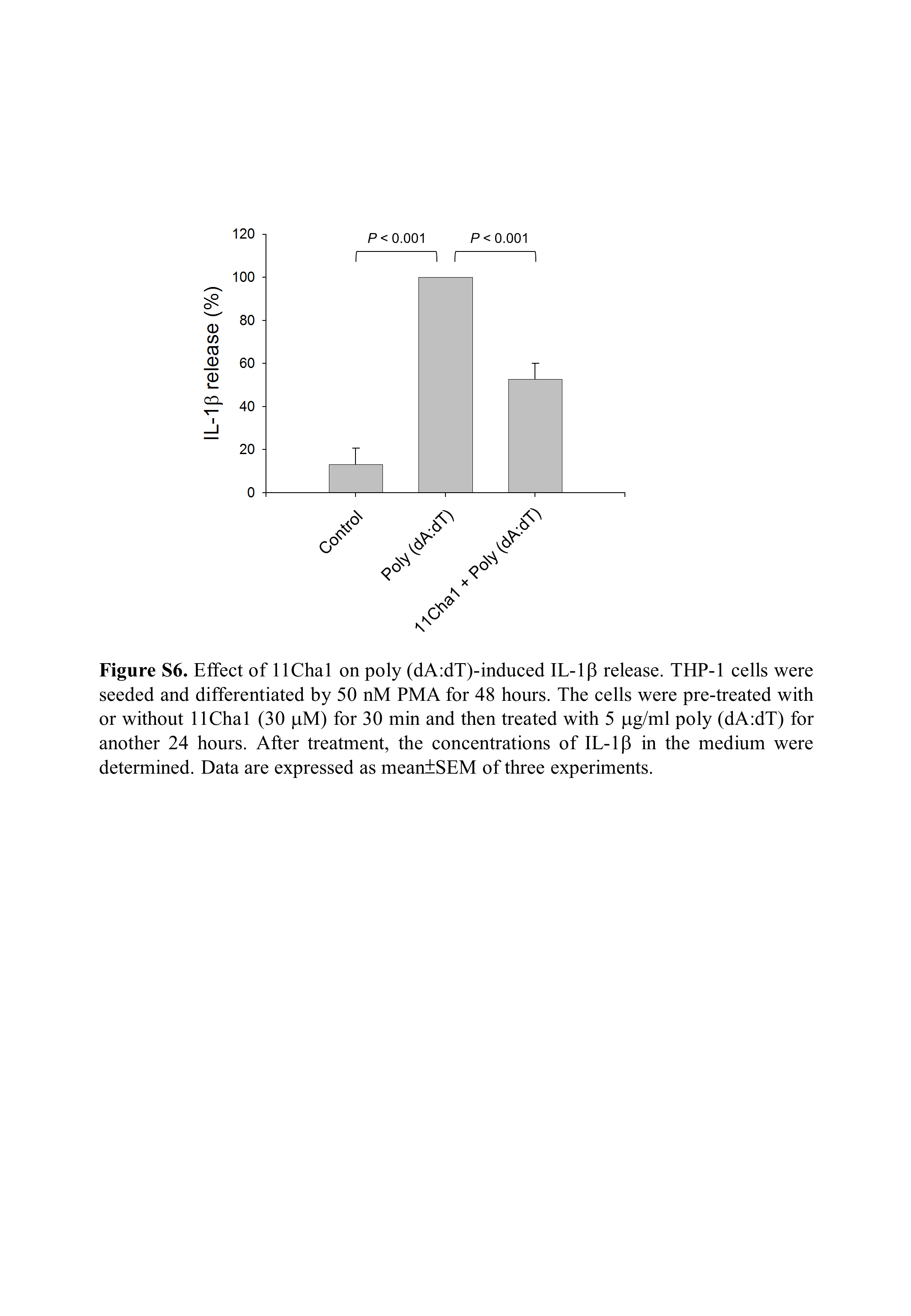

Supplement: Supplementary file 1 [file molecules-25-05960-s001.zip › Figure S6.tif]

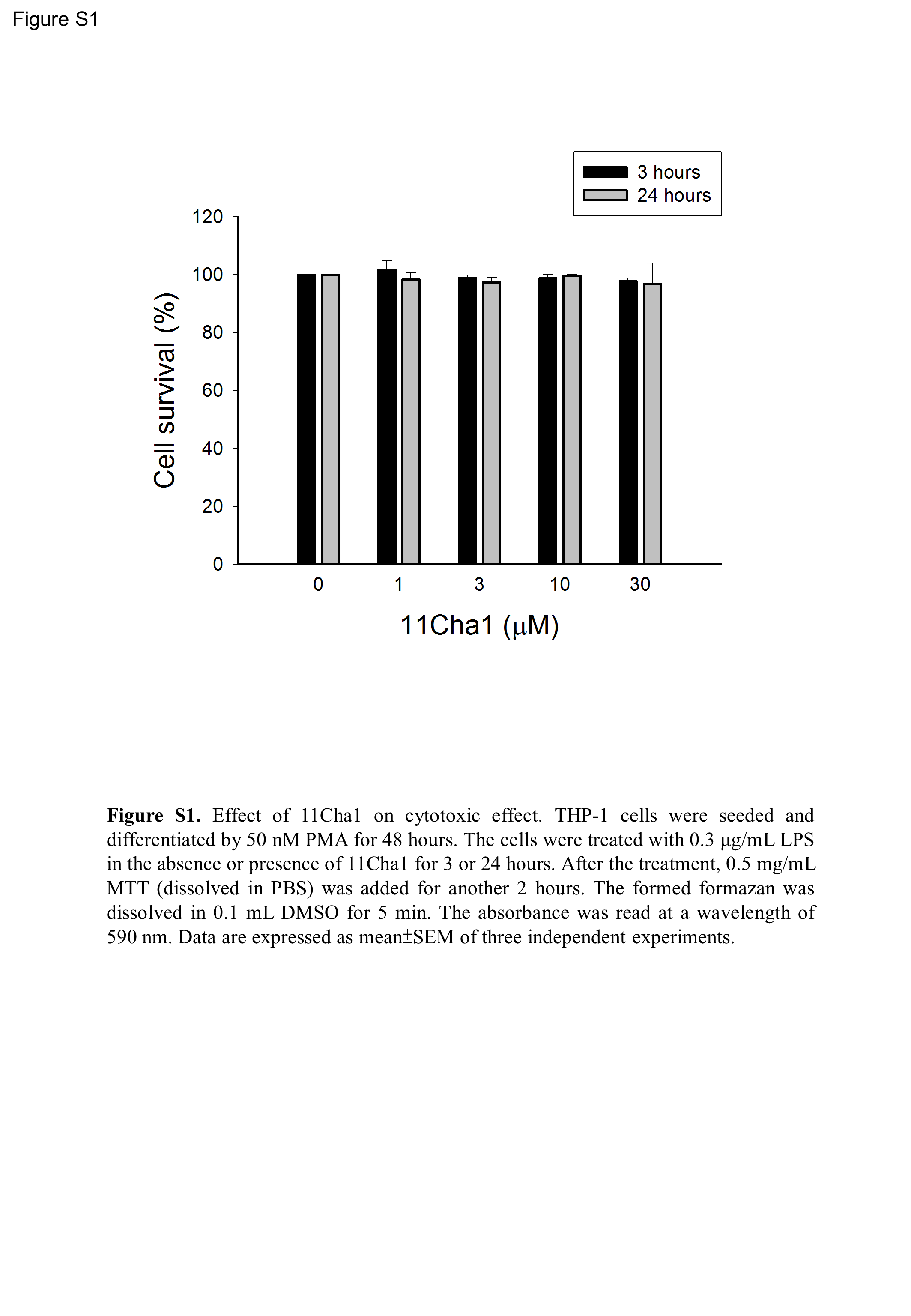

Supplement: Supplementary file 1 [file molecules-25-05960-s001.zip › Figure S1.tif]
